# Ccl2‐Induced Regulatory T Cells Balance Inflammation Through Macrophage Polarization During Liver Reconstitution

**DOI:** 10.1002/advs.202403849

**Published:** 2024-10-01

**Authors:** Rui Wang, Qing Liang, Qian Zhang, Shuchao Zhao, Yuxiang Lin, Bing Liu, Yinjiang Ma, Xiaoya Mai, Quanze Fu, Xiaorui Bao, Nan Wang, Binglin Chen, Peng Yan, Yongsheng Zhu, Kejia Wang

**Affiliations:** ^1^ Department of Pulmonary and Critical Care Medicine The First Affiliated Hospital of Xiamen University State Key Laboratory of Cellular Stress Biology Cancer Research Center School of Medicine Xiamen University Xiamen Fujian 361102 China; ^2^ Department of Pathology The Affiliated Hospital of Qingdao University Qingdao Shandong 266000 China; ^3^ National Institute for Data Science in Health and Medicine Xiamen University Xiamen Fujian 361102 China; ^4^ Department of Dermatology Zhongshan Hospital Xiamen University Xiamen Fujian 361102 China; ^5^ NHC Key Laboratory of Forensic Science National Biosafety Evidence Foundation College of Forensic Science Xi'an Jiaotong University Xi'an Shaanxi 710061 China

**Keywords:** inflammatory milieu, liver reconstitution, macrophage polarization, regulatory T cell, type 1 innate lymphoid cell

## Abstract

Inflammation is highlighted as an initial factor that helps orchestrate liver reconstitution. However, the precise mechanisms controlling inflammation during liver reconstitution have not been fully elucidated. In this study, a clear immune response is demonstrated during hepatic reconstitution. Inhibition of the hepatic inflammatory response retards liver regeneration. During this process, Ccl2 is primarily produced by type 1 innate lymphoid cells (ILC1s), and ILC1‐derived Ccl2 recruits peripheral ILC1s and regulatory T cells (Tregs) to the liver. Deletion of Ccl2 or Tregs exacerbates hepatic injury and inflammatory cytokine release, accelerating liver proliferation and regeneration. The adoption of Tregs and IL‐10 injection reversed these effects on hepatocyte regenerative proliferation. Additionally, Treg‐derived IL‐10 can directly induce macrophage polarization from M1 to M2, which alleviated macrophage‐secreted IL‐6 and TNF‐α and balanced the intrahepatic inflammatory milieu during liver reconstitution. This study reveals the capacity of Tregs to modulate the intrahepatic inflammatory milieu and liver reconstitution through IL‐10‐mediated macrophage polarization, providing a potential opportunity to improve hepatic inflammation and maintain homeostasis.

## Graphical abstract

This work reveals a crosstalk between various immune cells in hepatic inflammatory milieu after liver injury and teases out the network to orchestrate the regenerative process, which provides a novel immune‐associated insights into liver reconstitution. This ILC1/Ccl2/Treg/IL‐10/M2 axis may be an effective immunotherapeutic strategy for liver diseases.

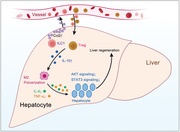

## 1 Introduction

The adult liver has a striking regenerative capacity following acute liver injury. Classic experiments in rodents have shown that after two‐thirds partial hepatectomy (PHx) or carbon tetrachloride (CCl_4_) treatment, the liver mass can be reconstituted.^[^ ^1^ ^]^ Hepatic inflammation, initiated by endogenous molecules from dead or dying cells, is set in place to trigger hepatic repair and promote the re‐establishment of homeostasis. However, an inflammatory response that is too intense or fails is nearly always accompanied by aberrant reconstitution or causes irreversible damage to the liver.^[^ ^2^ ^]^ Accumulating evidence demonstrates that the inflammatory pathways, including TNF‐α and IL‐6 signaling pathways, are robustly activated by inflammatory mediators such as cytokines, chemokines, and complements, which stimulate quiescent hepatocytes to enter the cell cycle.^[^ ^3^ ^]^ However, the communication between cytokines and immune cells, and the potential regulatory mechanisms to balance inflammation during liver reconstitution are largely unknown.

The liver is abundant in various immune cells. To date, nearly all immune cell subsets have been reported to be involved in liver reconstitution.^[^ ^4^ ^]^ No single cell population or cytokine has enough credibility to fully understand the hepatic regenerative process without considering interactions between distinct immune cells. Macrophages have long been identified as key players in the response to acute liver injury,^[^ ^5^ ^]^ as they produce IL‐6 and participate in liver regeneration by promoting hepatocyte proliferation via phosphorylation of STAT3 and activation of the phosphoinositide‐3 kinase (PI3K) pathway.^[^ ^6^, ^7^ ^]^ Recent evidence supports that acute liver injury results in the polarization of macrophages toward M1 (pro‐inflammatory) or M2 (anti‐inflammatory) phenotypes in response to extracellular stimuli.^[^ ^8^ ^]^ The balance between M1 and M2 macrophages plays a critical role in the resolution of the hepatic inflammatory response. Therefore, the termination of hepatic remodeling is largely determined by macrophage polarization.

Regulatory T cells (Tregs), a specialized T‐cell lineage, have a pivotal role in self‐tolerance and immune homeostasis. They contribute to the control and termination of the inflammatory response by producing anti‐inflammatory cytokines such as IL‐10, TGF‐β, and IL‐35.^[^ ^9^ ^]^ The X chromosome‐encoded transcription factor forkhead box P3 (Foxp3), identified as a lineage‐specific transcription factor of Tregs, is centrally involved in the development of Tregs and the maintenance of the Treg phenotype.^[^ ^10^ ^]^ Tregs may sense the initiation and progression of inflammation from the tissue microenvironment to adapt their suppressive functions.^[^ ^11^ ^]^ Accumulating evidence suggests that Tregs actively engage in tissue remodeling and repair in stressed or damaged organs. The enhanced frequency of intrahepatic Tregs has been reported in the injured liver to control liver inflammation, but whether they interact with other immune cell populations and their specific functions remains poorly understood.^[^ ^12^, ^13^ ^]^

The CCl_4_‐induced and two‐thirds PHx‐mediated mouse liver injury model was used to assess the interaction between different cell types in the injured liver. Here, we investigated the dynamics of cellular and molecular signatures in the injured liver regulated by type 1 innate lymphoid cells (ILC1s), Tregs, and macrophages, and how they together participate in controlling liver inflammation and modulating regenerative processes. We further identified the molecular mechanism by which Treg‐derived IL‐10 triggers M2 macrophage polarization during liver reconstitution.

## 2 Results

### 2.1 Immunosuppression Leads to Aberrant Hepatic Regeneration

**Figure 1:**
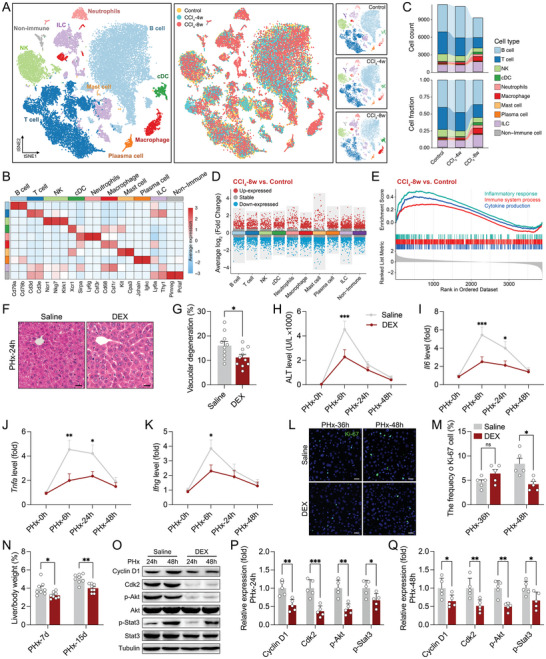
DEX inhibits inflammatory response and regenerative proliferation after liver injury. A) *t*SNE projection visualizing the total (left) and each time point (right, control, 4 and 8 weeks) of intrahepatic immune cells after CCl_4_ treatment (*n* = 3). B) Heatmap showing the marker genes among different cell subsets (*n* = 3). C) The cell count and fraction of indicated time point (*n* = 3). D) The expression of DEGs among ten major cell types (8 weeks versus control, *n* = 3). E) The enrichment score of DEGs in the inflammatory response and immune process (*n* = 3). F,G) Representative images of HE staining (F) and quantification of vacuolar degeneration (G) after saline or DEX treatment (*n* = 10, scale bar, 20 µm). H) The serum level of ALT at the indicated time point after PHx (*n* = 5). (I–K) qPCR analysis of the relative mRNA expression of *Il6* (I), *Tnfa* (J), *Ifng* (K) (*n* = 5). L,M) Representative fluorescence images (L) and quantification (M) of Ki‐67‐positive cells (*n* = 5, scale bar, 20 µm). N) The ratio of liver/body weight (*n* = 8). O–Q) Representative images of western blotting (O) and quantification of the protein expression 24 (P) or 48 h (Q) after PHx (*n* = 5). Data represent three to five independent experiments. Data are shown as the mean + SEM along with individual data points and were compared using unpaired Student's *t*‐test (G, P,Q) or two‐way ANOVA followed by Bonferroni's multiple comparisons test (H–K, M,N). ^*^ *p* < 0.05, ^**^ *p* < 0.01, ^***^ *p* < 0.001, ns indicates *p* > 0.05.

Two‐thirds PHx and CCl_4_‐induced hepatic injury mouse models were used to assess the liver immune microenvironment. To characterize the changes in immune cells following liver injury, we performed scRNA‐seq on CD45^+^ cells isolated from CCl_4_‐treated liver. *t*‐distributed stochastic neighbor embedding (*t*SNE) was used to reveal nine different immune cell subsets (**Figure** **1**A,B; Figure S1A and Table S1, Supporting Information). Indeed, liver injury induced an aberrant cell distribution (including upregulation of macrophages, innate lymphocyte, dendritic cell (cDC), and downregulation of T cells) and gene expression (Figure 1C,D; Figure S1B and Table S1, Supporting Information). Moreover, Go enrichment analysis indicated that the differentially expressed genes (DEGs) were associated with inflammatory responses and immune system processes (including TNF‐α and IL‐6 signaling) (Figure 1E; Table S1, Supporting Information).

To determine whether the inflammatory response is involved in liver reconstitution, dexamethasone (DEX) was used to suppress the hepatic inflammatory response after PHx (Figure S1C, Supporting Information). Notably, DEX significantly reduced vacuolar degeneration with lower serum alanine transaminase (ALT), *Il6*, *Tnfa*, and *Ifng* expression, suggesting alleviated hepatic inflammation (Figure 1F–K). Subsequently, livers with low inflammation showed decreased hepatocyte proliferation and liver‐to‐body weight ratio (Figure 1L–N). However, there was no significant change in cell apoptosis between saline‐ and DEX‐treated livers (Figure S1D, Supporting Information). Western blotting analysis showed reduced expressions of Cyclin D1, Cdk2, p‐Akt, and p‐Stat3 in the injured livers from DEX‐treated mice (Figure 1O–Q), which confirms the view that hepatic inflammation plays a critical role in initiating liver regeneration.^[^ ^14^ ^]^

### 2.2 Acute Liver Injury Enhances ILC1‐Derived Ccl2 Production

**Figure 2:**
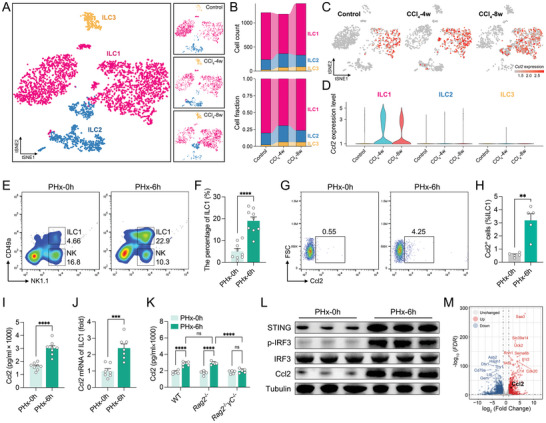
Liver injury leads to ILC1‐derived Ccl2 production. A) The ILC was re‐clustering from Figure 1A. Three ILC subtypes were identified based on clustering after CCl_4_ treatment (*n* = 3). B) The cell count and fraction of indicated time point after CCl_4_ treatment (*n* = 3). (C and D) The specificity C) and intensity D) of *Ccl2* expression in ILC subtypes (*n* = 3). E,F) Representative flow cytometry plots (E) and the statistical quantification (F) of hepatic ILC1s (CD49a^+^ NK1.1^+^) 6 h after PHx (*n* = 9). (G and H) Representative flow cytometry plots G) and percentage H) of the Ccl2^+^ cells in ILC1s (CD49a^+^ NK1.1^+^, gated from Figure 2E) (*n* = 5). I) The level of Ccl2 measured by ELISA (*n* = 8). J) qPCR analysis of the relative mRNA expression of *Ccl2* in the liver (*n* = 7). K) The level of Ccl2 measured by ELISA in WT, *Rag2* ^−/−^ (lack T and B cells), *Rag2* ^−/−^ *γC* ^−/−^ (lack T, B, ILC1s, and NK cells) mice (*n* = 5). L) 1×10^5^ ILC1s (CD49a^+^, NK1.1^+^) were isolated from the regenerative liver 6 h after PHx. The western blotting of cGAS‐STING (p‐IRF3, IRF3, and STING) and Ccl2 protein expression (*n* = 3). M) Volcano plots of DEGs in ILC1s. The red dots indicate upregulated genes. The blue dots indicate downregulated genes (*n* = 4). Data represent three to five independent experiments. Data are shown as the mean + SEM along with individual data points and were compared using unpaired Student's *t*‐test (F,I,J) or Mann–Whitney test (H) or two‐way ANOVA followed by Bonferroni's multiple comparisons test (K). ^**^ *p* < 0.01, ^***^ *p* < 0.001, ^****^ *p* < 0.0001.

Emerging evidence supports the notion that acute liver injury activates liver‐resident ILC1s, which promotes hepatocyte survival and protects the liver from damage.^[^ ^15^, ^16^, ^17^, ^18^ ^]^ To identify the role of ILC1 in liver injury, the profile of the ILC1 transcriptome was analyzed using scRNA‐seq data (**Figure** **2**A; Figure S2A–C and Table S2, Supporting Information). Notably, CCl_4_‐induced liver injury enhanced ILC1‐derived *Ccl2* and *Ccr2* expression, although there was no significant change in the ILC1 fraction (Figure 2B–D; Figure S2D,E, Supporting Information). Similarly, 2/3 PHx induced an increase in the ILC1 population and Ccl2 production (Figure 2E–H; Figure S2F, Supporting Information). ELISA and qPCR results confirmed hepatic Ccl2 enhancement after PHx (Figure 2I,J). The deficiency of ILC1s eliminated the effect of Ccl2 augmentation in the injured liver, indicating that the elevated Ccl2 was mainly produced by ILC1s (Figure 2K). To exclude the impact of NK cells, Ccl2 expression was detected in NK cells. There was no significant difference in Ccl2 expression in NK cells between 0 and 6 h after PHx (Figure S2G,H, Supporting Information). Together, these results indicate that the increased Ccl2 primarily comes from ILC1s.

Liver injury results in the production of massive amounts of danger‐associated molecular patterns (DAMPs) from dead cells, which activate the cGAS‐STING pathway.^[^ ^19^, ^20^ ^]^ Therefore, we isolated ILC1s from the regenerative liver. Interestingly, acute liver injury induced cGAS‐STING pathway activation with elevated Ccl2 expression in ILC1s. These results indicate that cGAS‐STING activation may contribute to Ccl2 secretion in ILC1s (Figure 2L). To further explore ILC1s gene expression changes after PHx, we sorted ILC1s from the regenerated liver (6 h after PHx) and performed bulk RNA‐seq. Acute liver injury led to a significantly different profile of gene expression in ILC1s (Figure 2M; Figure S3A and Table S3, Supporting Information). A range of cytokine and cytokine receptor genes (*Il17ra*, *Il13ra1*, *Ccl2*, *Ccl6*, *Ccl9*, *Ifngr1*, *Ccr1*, *Il1r1*, *Il1r2*, *Cxcr2*, *Il1b*, *Il18*, *Ccr2*, *Cxcl2*, *Ccr5*) were elevated after injury (Figure S3B and Table S3, Supporting Information). The DEGs were also enriched in the “inflammatory response” and “cytokine production” gene sets (Figure S3C,D and Table S3, Supporting Information), including PI3K/AKT and IL‐6/STAT3 signaling (Figure S3E,F and Table S3, Supporting Information).

### 2.3 Acute Liver Injury Leads to Ccl2‐Mediated ILC1s and Tregs Recruitment

**Figure 3:**
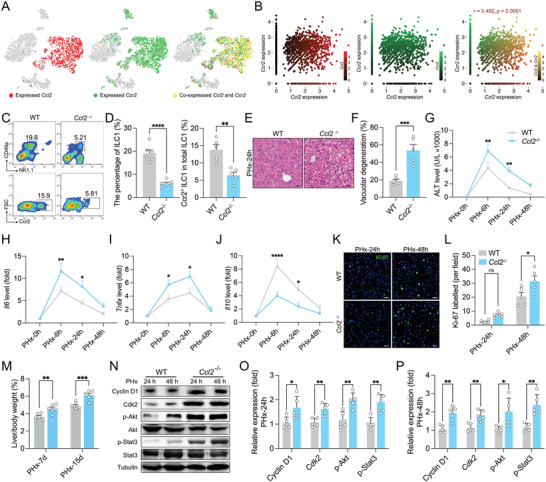
Deletion of Ccl2 results in deteriorated hepatic inflammation and enhanced hepatocyte proliferation in acutely injured livers. A) The scRNA‐seq data were analyzed from Figure 2A.The expression of *Ccl2* and *Ccr2* in *t*SNE plot. *Ccl2*‐positive cell colored by red. *Ccr2*‐positive cell colored by green. *Ccl2* and *Ccr2* coexpression colored by yellow (*n* = 3). B) Correlation analysis between the *Ccl2* and *Ccr2* expression (*n* = 3). C,D) Wildtype (WT) and *Ccl2* ^−/−^ mice were subjected to 2/3 PHx. Representative flow cytometry plots (C) and the statistical quantification (D) of hepatic ILC1s (CD49a^+^ NK1.1^+^) and Ccr2^+^ ILC1s 6 h after PHx (*n* = 5, 6). (E and F) Representative images of HE staining E) and quantification F) of vacuolar degeneration 24 h after PHx (*n* = 5, scale bar, 20 µm). G) Serum levels of ALT (*n* = 5). H–J) The relative mRNA expression of *Il6* (H), *Tnfa* (I) and *Il10* (J) (*n* = 5). K,L) Representative fluorescence images (K) and quantification (L) of Ki‐67‐positive cells (*n* = 5, scale bar, 20 µm). M) The ratio of liver/body weight (*n* = 6). N–P) Representative images of western blotting (N) and quantification (O,P) of the relative protein expression (*n* = 5). Data represent three to five independent experiments. The Spearman's correlation analysis was used in panel B. Data are shown as the mean + SEM along with individual data points and were compared using unpaired Student's *t*‐test (D,F,O,P) or two‐way ANOVA followed by Bonferroni's multiple comparisons test (G–J,L,M). ^*^ *p* < 0.05, ^**^ *p* < 0.01, ^***^ *p* < 0.001, ^****^ *p* < 0.0001, ns indicates *p* > 0.05.

Interestingly, few Ki‐67^+^ ILC1s and Tregs were found in the regenerative liver, suggesting that the expansion of these cells is due to recruitment (Figure S4A–D, Supporting Information). Previous studies also substantiate that the Ccl2/Ccr2 axis can recruit various immune cells (Tregs, monocytes, and NK cells) to specific organs.^[^ ^21^, ^22^ ^]^ As noted, scRNA‐seq data revealed a clear co‐expression of *Ccl2* and *Ccr2* in ILC1s, indicating that chronic liver injury led to ILC1s recruitment via autocrine mechanism (**Figure** **3**A,B). To determine whether the Ccl2/Ccr2 axis drives ILC1s homing to the injured liver, *Ccl2* gene knockout (*Ccl2* ^−/−^) mice were used to assess Ccl2‐mediated recruitment. As expected, the deletion of Ccl2 attenuated the recruitment of total and Ccr2^+^ ILC1s in response to acute liver injury (Figure 3C,D). Additionally, decreased Ccr2^+^ and total Tregs were detected in *Rag2* ^−/−^γC^−/‐^ and *Ccl2* ^−/‐^ livers after injury, suggesting that Ccl2 triggers Tregs homing (Figure S4E–L, Supporting Information). To verify whether ILC1‐derived Ccl2 mediates Treg recruitment, we created Foxp3^+^ cell‐specific Ccr2 knockout (*Ccr2* ^cKO^) mice (Figure S4M, Supporting Information). Although *Ccr2* knockout did not influence the maturation of Tregs (Figure S4N–Q, Supporting Information) or Tregs homing to the liver under steady state (Figure S4R–U, Supporting Information), *Ccr2* ^cKO^ mice exhibited lower Treg fraction in the injured liver (Figure S4V,W, Supporting Information). Taken together, these results indicate that acute liver injury triggers the expansion of Tregs due to ILC1‐secreted Ccl2 recruitment.

Interestingly, we found significantly enhanced vacuolar degeneration accompanied by elevated serum ALT after PHx in *Rag2* ^−/−^γC^−/−^, *Ccl2* ^−/‐^ or *Ccr2* ^cKO^ livers (Figure 3E–G; Figure S5A–F, Supporting Information). Additionally, *Ccl2* ^−/−^ or *Rag2* ^−/−^γC^−/−^mice exhibited increased *Il6* and *Tnfa* and decreased *Il10* expression in the injured liver, demonstrating an aggravated inflammatory response during liver regeneration (Figure 3H–J; Figure S5G,H, Supporting Information). Indeed, deficiency of the Ccr2/Ccl2 axis promoted hepatocyte regenerative proliferation, resulting in a notable increase in liver‐to‐body weight ratio (Figure 3K–M; Figure S5I–L, Supporting Information). Notably, western blotting also revealed increased expression of Cyclin D1, Cdk2, p‐Akt, and p‐Stat3 in the injured livers of *Ccl2* ^−/−^ mice (Figure 3N–P). These data suggest that Ccl2 modulates cell‐cycle progression and inflammatory signaling.

### 2.4 Tregs are Involved in Liver Regeneration and Development

**Figure 4:**
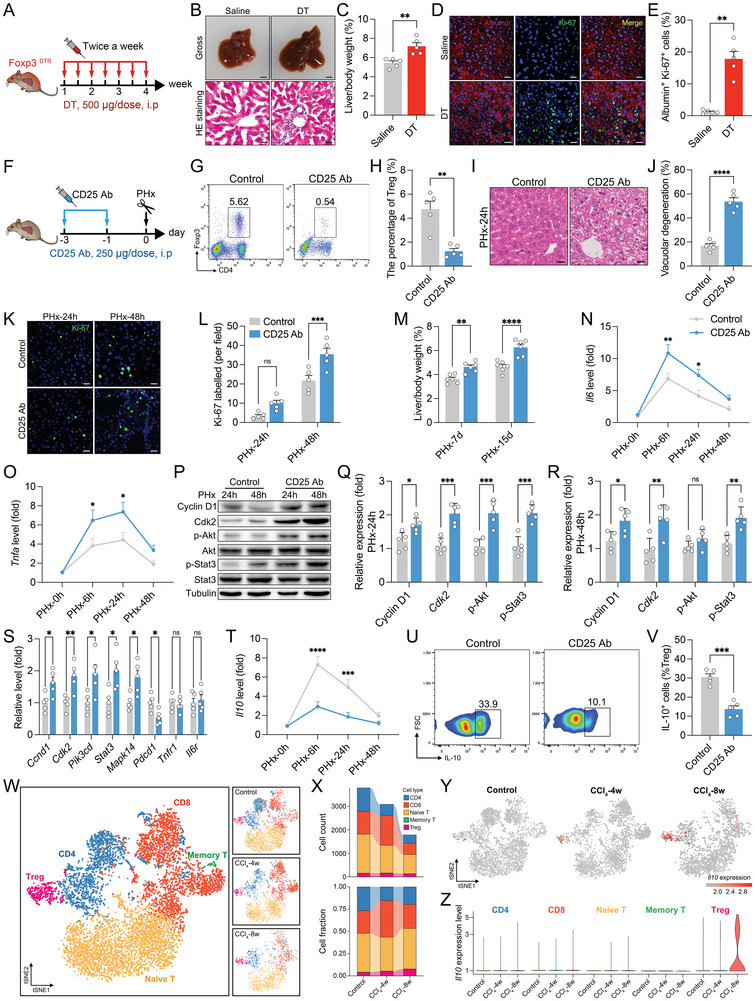
Treg balance hepatic inflammatory response to control liver regeneration and development. A) The liver tissue from Foxp3^DTR^ mice after 4 weeks of DT injection. Flow chart of DT administration. B,C) Representative images of histology (B) and quantification (C) of liver/body weight ratio (*n* = 5, gross scale bar, 2.5 mm; HE staining scale bar, 20 µm). D,E) Representative fluorescence images (D) and quantification (E) of Ki‐67‐positive cells (*n* = 5, scale bar, 20 µm). F) Flow chart of anti‐CD25 antibody administration. G,H) Representative flow cytometry plots (G) and the statistical quantification (H) of hepatic Tregs (CD4^+^ Foxp3^+^) (*n* = 5). I,J) Representative images of HE staining (I) and quantification (J) of vacuolar degeneration (*n* = 5, scale bar, 20 µm). K,L) Representative fluorescence images (K) and quantification (L) of Ki‐67‐positive cells (*n* = 5, scale bar, 20 µm). M) The ratio of liver/body weight (*n* = 6). N,O) The relative mRNA expression of *Il6* (N) and *Tnfa* (O) (*n* = 5). P–R) Representative images of immunoblotting (P) and quantification (Q,R) of the relative protein expression (*n* = 5). S) The relative mRNA expression by qPCR 6 h after PHx (*n* = 5). T) The relative mRNA expression of *Il10* (*n* = 5). U,V) Representative flow cytometry plots (U) and percentage (V) of IL‐10^+^ cells in Tregs (CD4^+^ Foxp3^+^, gated from Figure 4G) 6 h after PHx (*n* = 5). W) The T cells were re‐clustering from Figure 1A. Five T cell subtypes were identified based on clustering after CCl_4_ treatment (*n* = 3). X) The cell count and fraction of indicated time point after CCl_4_ treatment (*n* = 3). Y,Z) The specificity (Y) and intensity (Z) of *Il10* expression in T cell subtypes (*n* = 3). Data represent three to five independent experiments. Data are shown as the mean + SEM along with individual data points and were compared using unpaired Student's *t*‐test (C,H,J, Q–S,V) or Mann–Whitney test (E) or two‐way ANOVA followed by Bonferroni's multiple comparisons test (L–O,T). ^*^ *p* < 0.05, ^**^ *p* < 0.01, ^***^ *p* < 0.001, ^****^ *p* < 0.0001, ns indicates *p* > 0.05.

As mentioned above, the Ccr2/Ccl2 axis recruits peripheral Tregs to the injured liver. To explore the role of Tregs in hepatocyte proliferation, we used scurfy mice (*Foxp3* ^−/y^), Foxp3^DTR^ knock‐in mice, and CD25 neutralizing antibody (CD25 Ab)‐injected mice to determine whether Tregs affect hepatocyte proliferation during liver development or regeneration. Hepatocytes undergo rapid proliferation within the first month after birth. Notably, Treg deficiency aggravated hepatic vacuolar degeneration (Figure S5M,N, Supporting Information) and increased hepatocyte proliferation (Figure S5O,P, Supporting Information). We also observed an increase in the liver‐to‐body weight ratio independent of body weight changes (Figure S5Q,R, Supporting Information). Moreover, we found a swollen liver with increased immune cell infiltration after 4 weeks of DT administration in Foxp3^DTR^ knock‐in mice (**Figure** **4**A–C). Additionally, DT administration led to increased hepatocyte proliferation (Figure 4D,E). CD25 Ab administration also resulted in a decreased percentage of Tregs (Figure 4F–H), with similar changes observed during liver regeneration (Figure 4I–M). These observations further support that Tregs act as suppressors of hepatic inflammatory response both in physiological conditions and in liver injury.

### 2.5 Tregs Balance Inflammation to Control Regenerative Progress

Similarly, Tregs deficiency led to remarkably elevated expression levels of *Il6* and *Tnfa* (Figure 4N,O). qPCR and Western blotting identified significantly activated AKT and STAT3 signaling in CD25 Ab‐treated mouse livers, demonstrating enhanced inflammation and proliferation signals in Treg‐deficient livers (Figure 4P–S). We then investigated the potential mechanism by which Tregs regulate liver regeneration. Accumulating evidence supports the notion that Tregs play a pivotal role in controlling the inflammatory response via the production of anti‐inflammatory cytokines (IL‐10, TGF‐β, and IL‐35).^[^ ^10^, ^23^ ^]^ Indeed, blocking CD25 significantly reduced *Il10* levels during liver regeneration, while CD25 Ab administration did not affect the expression of *Tgfb* and *Il35* in the liver (Figure 4T; Figure S5S,T, Supporting Information). Flow cytometry confirmed a decrease in the level of IL‐10 produced by Tregs (Figure 4U,V). Moreover, the gene profile of various T cell subsets (Naïve T, Memory T, Th17, CTL, Treg) was identified by scRNA‐seq data (Figure 4W; Figure S6A–E and Tables S1 and S2, Supporting Information). These results also revealed an increased Treg fraction and Treg‐derived IL‐10 production during liver injury (Figure 4X–Z).

**Figure 5:**
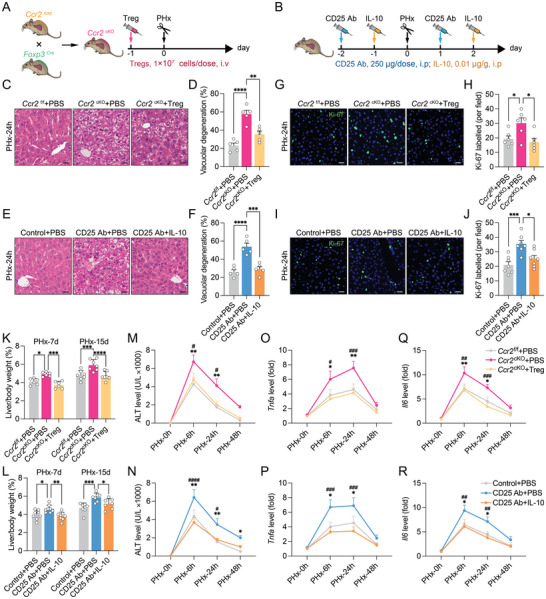
Treg‐derived IL‐10 controls regenerative proliferation. A,B) Flow chart of Treg adoption (A) or anti‐CD25 antibody and IL‐10 administration (B). C–F) Representative images of HE staining (C,E) and quantification (D,F) of vacuolar degeneration 24 h after PHx (*n* = 5, scale bar, 20 µm). G–J) Representative images (G,I) and quantification (H,J) of Ki‐67 positive cells 24 h after PHx (*n* = 6, 7, scale bar, 20 µm). K,L) The ratio of liver/body weight (*n* = 6–8). M,N) Serum levels of ALT (*n* = 5). O–R) The relative mRNA expression of *Tnfa* (O,P) and *Il6* (Q,R) (*n* = 5). Data represent three independent experiments. Data are shown as the mean + SEM along with individual data points and were compared using one‐way ANOVA followed by Bonferroni's multiple comparisons test (D,F,H,J) or two‐way ANOVA followed by Bonferroni's multiple comparisons test (K–R). ^*^ *p* < 0.05, ^**^ *p* < 0.01, ^***^ *p* < 0.001, ^****^ *p* < 0.0001, comparison between the 2 indicated groups, or comparison between the *Ccr2* ^f/f^ + PBS and *Ccr2* ^cKO^ + PBS groups, or comparison between the Control+PBS and CD25Ab + PBS groups; ^#^ *p* < 0.05, ^##^ *p* < 0.01, ^###^ *p* < 0.001, ^####^ *p* < 0.0001, comparison between the *Ccr2* ^cKO^ + PBS and *Ccr2* ^cKO^ + Treg, or comparison between the CD25Ab + PBS and CD25Ab + IL‐10 groups.

To test whether Tregs could directly impact the proliferation of hepatocytes, we isolated splenic Tregs from *Foxp3* ^YFP‐cre^ mice and co‐cultured the mouse hepatic cell line NCTC1469 with Tregs/IL‐10 in vitro (Figure S7A, Supporting Information). Interestingly, CFSE analysis revealed that Tregs or IL‐10 did not directly affect hepatocyte proliferation (Figure S7B, Supporting Information). Subsequently, we transfused Tregs and IL‐10 into *Ccr2* ^cKO^ and CD25 Ab‐treated mice according to the regimen (**Figure** **5**A,B). Restoration of Tregs and IL‐10 prevented excessive vacuolar degeneration and liver injury during liver regeneration (Figure 5C–F), which reduced hepatocyte proliferation (Figure 5G–J) and the liver‐to‐body weight ratio (Figure 5K,L). Adoptive transfer of Tregs or IL‐10 treatment also alleviated liver injury (Figure 5M,N) and suppressed hepatic *Il6* and *Tnfa* expression (Figure 5O–R), indicating that Tregs could inhibit the inflammatory response and regenerative proliferation via IL‐10 production.

### 2.6 Treg‐Derived IL‐10 Converts Macrophage Polarization from M1 to M2

**Figure 6:**
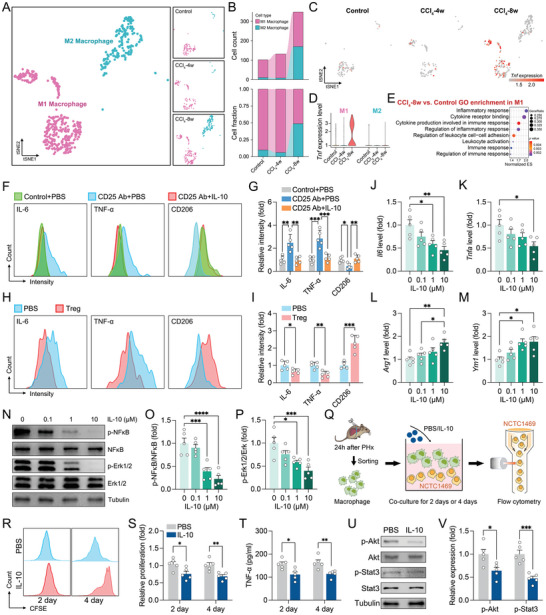
IL‐10 forces M2 macrophage polarization to regulate hepatocyte proliferation. A) The macrophages were re‐clustering from Figure 1A. Two macrophage subtypes were identified based on clustering after CCl_4_ treatment (*n* = 3). B) The cell count and fraction of indicated time point after CCl_4_ treatment (*n* = 3). C, D) The specificity (C) and intensity (D) of *Tnf* expression in T cell subtypes (*n* = 3). E) Go enrichment of DEGs in M1 (*n* = 3). F–I) Representative histogram plots (F and H) and quantification (G and I) of the fluorescence intensity of macrophage (CD11b^+^)‐gated IL‐6, TNF‐α and CD206 24 h after PHx (*n* = 5). J–M) Macrophages were isolated and incubated with IL‐10 for 48 h. The relative mRNA expression by qPCR (*n* = 5). N–P) Representative images of western blotting (N) and quantification (O, P) of the relative protein expression (*n* = 5). Q) Flow chart of co‐culture procedure in vitro. R, S) NCTC1469 cells were labeled with CFSE and co‐cultured with macrophages (isolated from the injured liver) containing PBS/IL‐10 for 2 days or 4. Representative histogram plots (R) and quantification (S) of the proliferation ratio of NCTC1469 cells (*n* = 5). T) The expression levels of TNF‐α in the culture supernatants were measured by ELISA (*n* = 5). U, V) Representative images of immunoblotting (U) and quantification (V) of the relative protein expression in NCTC1469 cells (*n* = 5). Data represent three independent experiments. Data are shown as the mean + SEM along with individual data points and were compared using unpaired Student's *t*‐test (I, V) or one‐way ANOVA followed by Bonferroni's multiple comparisons test (G, J–M, and O–P) or two‐way ANOVA followed by Bonferroni's multiple comparisons test (S, T). ^*^ *p* < 0.05, ^**^ *p* < 0.01, ^***^ *p* < 0.001, ^****^ *p* < 0.0001.

As mentioned above, Treg‐derived IL‐10 did not directly influence hepatocyte proliferation. Recent studies support the notion that IL‐10 induces a dominant immunosuppressive M2 macrophage phenotype via polarization.^[^ ^24^, ^25^ ^]^ To explore whether Treg‐derived IL‐10 modulates proliferation by controlling macrophage polarization, macrophage subsets were analyzed using scRNA‐seq data (**Figure** **6**A; Figure S8A–C and Table S2, Supporting Information). As expected, liver injury induced an expansion of M2 macrophages with elevated M1 macrophage‐derived *Tnf* levels (Figure 6B–D). In contrast, *Ccl2* expression was rarely detected in both M1 and M2 macrophages (Figure S8D, Supporting Information). The DEGs of M1 macrophages were enriched in the “inflammatory response” and “immune response” categories (Figure 6E; Table S4, Supporting Information). Interestingly, *Il10ra* and *Tnf* were co‐expressed in M1 macrophages, indicating that IL‐10 could interact with M1 macrophages to trigger M1 transformation during liver injury (Figure S8E–H, Supporting Information). CellChat analysis also indicated closer interactions between ILC1, macrophages, and Tregs (Figure S8I, Supporting Information).

Subsequently, adoptive transfer of Tregs or IL‐10 injection induced M1 to M2 polarization in total macrophages (CD11b^+^), Kupffer cells (CD11b^+^ F4/80^high^), and monocyte‐derived macrophages (CD11b^+^ F4/80^low^) (Figure 6F–I; Figure S9A–C, Supporting Information), although no significant change in the total number of macrophages was observed after treatment (Figure S9D–G, Supporting Information). Moreover, deficiency of Ccl2 and ILC1s resulted in elevated TNF‐α and reduced CD206 in macrophages, demonstrating that Ccl2‐induced Tregs convert macrophage polarization from M1 to M2 (Figure S9H–K, Supporting Information). To explore whether Ccl2 regulates M1/M2 polarization, macrophages were isolated from the injured liver (6 h after PHx) and incubated with Ccl2 for 48 h. Interestingly, no difference in IL‐6, TNF‐α, or CD206 levels was found after treatment, suggesting that Ccl2's influence on M1/M2 polarization relies on Tregs (Figure S9L,M, Supporting Information). Furthermore, co‐culture with macrophages and Tregs significantly induced M2 macrophage transformation (Figure S9N,O, Supporting Information). Additionally, IL‐10 treatment inhibited the expression of *Il6* and *Tnfa* and boosted the expression of *Arg1* and *Ym1* in a dose‐dependent manner (Figure 6J–M), indicating that Treg‐derived IL‐10 directly promotes M1 to M2 polarization in macrophages independently of direct interaction. IL‐10 treatment also inactivated both the MAPK and NF‐κB signaling pathways (important for macrophage polarization) in macrophages, as indicated by increased protein levels of p‐Erk1/2 and p‐NFκB (Figure 6N–P). NCTC1469 cells were co‐cultured with isolated macrophages and IL‐10 to assess the effect of macrophage polarization on hepatocyte proliferation (Figure 6Q). Co‐culture with macrophages and IL‐10 significantly impeded hepatocyte proliferation (Figure 6R,S), which was accompanied by decreased TNF‐α levels and reduced p‐Erk1/2 and p‐NFκB levels (Figure 6T–V). These findings indicate that by controlling M2 polarization, IL‐10 modulates the hepatic immune milieu and liver regeneration.

## 3 Conclusion

Immune activation and inflammatory surges are key features of various acute and chronic liver injuries. Unresolved inflammation acts as a detrimental factor in many liver diseases (hepatitis, cirrhosis, and cancer) by exacerbating liver damage.^[^ ^1^, ^26^ ^]^ Therefore, identifying the key factors that regulate the transition between healthy and pathogenic inflammation is critical for developing novel therapeutic strategies against inflammation‐related liver diseases. Several subsets of innate (including macrophages, NK, NKT, γδT, ILC, and cDC) and adaptive immune cells (including T and B cells) affect the hepatic immune microenvironment.^[^ ^1^, ^4^ ^]^ Previous studies have often focused on the function of specific cell types during liver regeneration. However, the interaction between distinct cell types to ensure an efficient and effective regeneration process remains unknown. Our work illustrates the network between immune cells that balances the inflammatory milieu during liver regeneration.

Type 1 innate lymphoid cells are tissue‐resident innate lymphocytes that regulate tissue inflammation and homeostasis.^[^ ^15^, ^16^, ^17^, ^18^ ^]^ ILC1s rapidly produce specific cytokines in response to local inflammation.^[^ ^27^ ^]^ In the liver, ILC1s are the most predominant ILC subset, except for NK cells. However, the pathophysiological role of hepatic ILC1s remains incompletely explored. A recent study highlighted the critical role of liver ILC1s in tissue protection during acute liver injury.^[^ ^15^ ^]^ Indeed, our results support the conclusion that acute liver injury induces ILC1s recruitment. Accumulating evidence indicates that Ly6c^high^ monocytes extravasate into the injured liver in a Ccl2/Ccr2‐dependent manner.^[^ ^1^, ^28^ ^]^ However, the origin of Ccl2 in the injured liver remains unknown. In this study, we demonstrate that ILC1s are primarily responsible for Ccl2 production in response to liver injury, which not only triggers monocyte recruitment but also drives ILC1s and Tregs homing to the liver.

Tregs, which belong to a subset of CD4^+^ T cells, regulate other immune cells in a dominant‐negative manner via the production of immunosuppressive cytokines such as IL‐10, TGF‐β, and IL‐35. The current study indicates Ccl2‐dependent Treg expansion in acutely injured livers, consistent with previous studies.^[^ ^13^, ^29^ ^]^ As noted, Tregs exert protective effects against acute liver injury, thereby promoting liver regeneration. In line with findings from other studies, chronic inflammation results in an increased proportion of Tregs in the liver via the IL‐33/ST2 axis.^[^ ^12^ ^]^ Furthermore, we demonstrated that Treg‐derived IL‐10 plays an immunosuppressive role in maintaining hepatic inflammation, which regulates the liver regeneration process. This finding supports the notion that IL‐10 exerts a negative role in modulating liver regeneration by inhibiting the inflammatory response and tempering hepatic IL‐6/STAT3 signaling activation.^[^ ^30^ ^]^

Macrophages are indispensable for the hepatic inflammatory milieu, fibrosis, and resolution, switching their phenotype from pro‐inflammatory to restorative. Additionally, macrophages are highlighted as key regulators that help orchestrate liver regeneration.^[^ ^1^ ^]^ It is believed that macrophage‐derived cytokines (TNF‐α and IL‐6) are essential for liver regeneration after PHx.^[^ ^31^, ^32^ ^]^ Two major classes of macrophages have been identified in the injured liver. The proportion of M1/M2 cells depends on their adaptive response to various stimuli. The heterogeneity or plasticity of macrophages exhibits different effects on liver regeneration.^[^ ^1^ ^]^ A recent study convincingly showed that macrophage polarization is implicated in inflammation resolution and liver regeneration. Ccl5 deficiency‐mediated M2 macrophage polarization accelerates liver repair by improving the inflammatory microenvironment.^[^ ^8^ ^]^ Here, we identified that IL‐10 shifts macrophage polarization from M1 to M2, which reshapes the hepatic inflammatory state and controls liver regeneration. These findings support the notion that the STAT3/IL‐10/IL‐6 axis is an important regulator of macrophage efferocytosis, survival, and phenotypic conversion,^[^ ^33^ ^]^ verifying that the balance of hepatic macrophage polarization is finely modulated by Treg recruitment and IL‐10 production.

In summary, our study reveals a network of distinct immune cell interactions that regulate the inflammatory microenvironment during liver regeneration. ILC1‐derived Ccl2 drives ILC1s and Tregs homing to the injured liver via paracrine and autocrine mechanisms. This chemotactic effect and subsequent IL‐10 production play a key role in modulating hepatic inflammation and regeneration. Our work also illustrates the mechanism of macrophage polarization and clarifies the effect of macrophage phenotype on liver regeneration. This ILC1/Ccl2/Treg/IL‐10/M2 axis may provide new immune‐associated insights into liver injury and repair.

## 4 Experimental Section

### Mice

*Rag2* ^−/−^ (Cat# 033562), *Rag2* ^−/−^γC^−/−^ (Cat# 014593), *Ccl2* ^−/−^ (Cat# 004434), *Foxp3* ^YFP‐Cre^ (Cat# 016959), Scurfy mice (*Foxp3* ^−/y^, Cat# 006775), Foxp3^DTR^ knock‐in mice (Cat# 016958) were purchased from The Jackson Laboratory (Bar Harbour, USA). *Ccr2* ^cKO^ mice were generated by crossing *Ccr2* ^loxP^ (Gempharmatech, Nanjing, China) mice with *Foxp3* ^YFP‐Cre^ mice. For Tregs ablation, 6‐week‐old Foxp3^DTR^ mice were intraperitoneally injected twice a week with 500 ng dose^−1^ of diphtheria toxin (DT, List Labs, California, USA) for 3 weeks. All mice were housed and maintained under specific pathogen‐free (SPF) conditions. All animal experiments, housing, and feeding conditions were approved by the Institutional Ethical Commission for Animal Research at Xiamen University (XMULAC20190061).

### Animal Treatment and Single‐Cell RNA Sequencing (scRNA‐seq)

Mice (8‐ to 12 weeks old) were subjected to 2/3 partial hepatectomy (PHx) and carbon tetrachloride (CCl_4_, Shanghai Acmec Biochemical Technology Co., Ltd, Shanghai, China)‐induced hepatic injury as described previously.^[^ ^34^, ^35^ ^]^ Mice were given an intraperitoneal injection of 2 mg kg^−1^ dexamethasone (DEX, Shanghai Shyndec Pharmaceutical Co., Ltd, Shanghai, China) once a day for three days before PHx. CD25 neutralizing antibody (250 µg/dose, clone PC‐61.5, BioXcell, New Hampshire, USA) and IL‐10 (0.01 µg/g, Novoprotein, Suzhou, China) were injected intraperitoneally twice a week before PHx. The intrahepatic immune cells were isolated according to the previous study. Single CD45^+^ cells were sorted from untreated, 4‐week, and 8‐week CCl_4_‐treated liver samples by fluorescence‐activated cell sorting (FACS), followed by droplet‐based scRNA‐seq on a 10X Genomics Chromium chip (10X Genomics, Pleasanton, USA). The 10X Genomics Single Cell v2 kit was used for reverse transcription and library preparation. Subsequently, the libraries were sequenced on an Illumina HiSeq 4000 (Illumina, San Diego, USA).

### Preprocessing and Integrating scRNA‐seq Data

In this study, single‐cell RNA sequencing (scRNA‐seq) data were preprocessed and integrated using the well‐established Seurat R package (version 4.1.1). Initially, genes detected in fewer than three cells were filtered out to ensure data quality. Subsequently, low‐quality cells characterized by a limited number of measured genes (< 200 genes) were excluded and potential doublets exhibiting more than 6 000 genes. Additionally, cells with mitochondrial gene expression constituting more than 20% of the total gene counts were removed to further enhance data reliability. To integrate the single‐cell data derived from mice subjected to three distinct experimental conditions, the Seurat package was employed, selecting 2 000 features for cell integration. This approach resulted in a comprehensive dataset comprising 32256 cells in total, enabling to investigate cellular heterogeneity and expression patterns across different experimental groups.

### Unsupervised Clustering and Cell Identification

The conventional workflow in the Seurat R package was employed to perform dimensionality reduction and unsupervised clustering on each scRNA‐seq dataset. The optimal cluster resolution value was determined using the cluster R package (version 0.5.0). To identify cell types and subpopulations, nine cell types were initially distinguished based on the expression of canonical marker genes. Specifically, B cells were identified by positive expression of *Cd79a* and *Cd79b*, while T cells were recognized by high expression of *Cd3d* and *Cd3e*. Natural killer (NK) cells were marked by *Ncr1*, *Nkg7*, and *Klrk1* expression, and classical dendritic cells (cDCs) were differentiated by *Xcr1* and *Sirpa* expression. Neutrophils were characterized by *Ly6g* and *Csf3r* expression, macrophages by *Cd68* and *Csf1r*, mast cells by *Kit* and *Cpa3*, plasma cells by *Jchain* and *Igkc*, and innate lymphoid cells (ILCs) by *Ly6a* and *Thy1*. Non‐immune cells were identified by high expression of *Pimreg* and *Pclaf*. To further delineate T cell and ILC subpopulations, the same conventional pipeline was applied after subsetting all T cells and ILCs separately. T cells were classified into CD4 T cells (*Cd4*, *Icos*), CD8 T cells (*Cd8a*, *Gzmk*), naïve T cells (*Lef1*, *Tcf7*), memory T cells (*Il7r*, *Ms4a6b*), and regulatory T cells (Tregs; *Cd5*, *Foxp3*, *Ilzf2*). Regarding ILCs, ILC1, ILC2, and ILC3 were distinguished based on canonical markers. ILC1 expressed *Ncr1*, *Gzma*, and *Plek*, ILC2 expressed *Gata3*, *Rora* and *Nr4a2*, and ILC3 expressed Th17‐associated markers, such as *Il17a*, *Il17f*, and *Rorc*. Moreover, macrophages were classified into M1 macrophages (*Tnf*, *Cd86*, *Il1b*) and M2 macrophages (*Bst2*, *Il4ra*).

### Cell–Cell Communication Analysis

The intercellular communication between ILCs, T cell, and macrophages was analyzed using the CellChat R package (version 1.6.1).

### Differential Expression Gene Detection

Differentially expressed genes (DEGs) among each cell cluster between groups were identified using the “FindMarkers” function from the Seurat R package with the Wilcoxon rank‐sum test algorithm. Genes with a *q* value < 0.05, log2|Fold Change| > 0.25, and min. pct > 0.1 were considered differentially expressed.

### Pathway Enrichment Analysis

To investigate the biological significance of the DEGs identified in the previous step, a Gene Ontology (GO) pathway enrichment analysis was performed, focusing on overrepresented immunity‐related and inflammatory‐associated biological processes within the context of the DEGs. The clusterProfiler R package (version 4.2.2) was employed to conduct the enrichment analysis, utilizing a hypergeometric test to calculate the statistical significance of the overrepresented GO terms.

### Bulk RNA Sequencing (RNA‐seq) and Quantitative PCR (qPCR)

Total RNA was isolated using TRIeasy LS Total RNA Extraction Reagent (Yeasen Biotechnology, Shanghai, China). cDNA was synthesized by chloroform extraction and isopropanol precipitation as described previously.^[^ ^36^ ^]^ A Transcriptor First Strand cDNA Synthesis Kit (Yeasen Biotechnology) was used for cDNA synthesis and strand‐specific library preparation. The libraries were sequenced on an Illumina NovaSeq 6 000 instrument (Illumina, San Diego, USA) using the PE150 strategy. R package DESeq2 was used to compare the DEGs. Genes with a *q* value < 0.05 and log2|Fold Change| > 1 were deemed differentially expressed. Gene Ontology (GO) and Kyoto Encyclopedia of Genes and Genomes (KEGG) analyses were performed using the R package ClusterProfiler.

To detect mRNA expression, qPCR was performed using SYBR Green PCR mix (Yeasen Biotechnology) and conducted with the qTOWER3G touch (Analytik Jena, Jena, Germany). Relative gene expression levels of mRNA were normalized against *Gapdh* and calculated using the 2^−ΔΔCt^ method. Primer sequences are shown in Table S5 (Supporting Information).

### Immune Cell Isolation and Flow Cytometry

Isolation of intrahepatic immune cells was conducted as described in the previous study. Briefly, the excised liver tissue was minced into small pieces and gently triturated through a 75 µm gauge stainless steel mesh. The suspension was centrifuged at 850 × g for 30 min with 37.5% Percoll (Solarbio Life Sciences, Beijing, China) in Hank's balanced salt solution (HBSS, Solarbio Life Sciences). The single suspension was then stained with fluorescently conjugated antibodies against PE‐CD3 (17A2), APC‐CD3 (17A2), PerCP‐CD45 (30‐F11), APC‐CD49a (clone: HMα1), APC‐Ccr2 (SA203G11), FITC‐NK1.1 (S17016D), PE‐Ccl2 (2H5), PE‐Foxp3 (clone: 150D), FITC‐CD4 (clone: GK1.5), FITC‐F4/80 (BM8), PE‐CD11b (M1/70), APC‐IL‐6 (MP5‐20F3), APC‐Ki‐67 (16A8), PerCP/Cyanine5.5‐lL‐10 (JES5‐16E3), PE‐TNF‐α (MP6‐XT22) and PE‐CD206 (C068C2) from BioLegend. The cells were fixed and permeabilized using the Foxp3/Transcription Factor Staining Buffer Set (eBioscience, Shanghai, China) for cytokine detection. Briefly, the cells were treated with Fixation/Permeabilization Solution for 30 min at 4 °C, avoiding light, followed by washing with Permeabilization Buffer. Subsequently, the cytokine antibodies were used for staining at 4 °C for 45 min. The stained cells were examined using a BD Aria III machine and analyzed with FlowJo software (version 10.6.2, BD, Ashland, USA).

### Cell Culture

Splenic Tregs (CD4^+^YFP^+^) from *Foxp3* ^YFP‐cre^ mice were sorted and cultured in RPMI supplemented with 10% FBS (Yeasen, Shanghai, China). The isolated Tregs were incubated with 1000 IU IL‐2 (Novoprotein), 100 ng mL^−1^ PMA (Sigma, Shanghai, China), and 500 ng mL^−1^ ionomycin (Sigma) together for 72 h. For Treg adoption, a total of 1×10^7^ Tregs were intravenously infused into each mouse 2 days before PHx. Mouse NCTC1469 normal liver cells (American Type Culture Collection, ATCC) were cultured in DMEM (Solarbio Life Sciences) supplemented with 10% FBS (Cegrogen Biotech, Eupen, Belgium). To detect cell proliferation, NCTC1469 cells were labeled with 0.2 µM CFSE (Invitrogen, Shanghai, China) and analysed by flow cytometry. All cells were cultured at 37 °C in a humidified atmosphere with 5% CO_2_.

For co‐culture transwell experiments, 1 × 10^5^ macrophages (CD11b^+^, isolated from PHx‐6 h liver) were seeded in the lower chamber and co‐cultured with 1 × 10^5^ Tregs (CD4^+^YFP^+^, isolated from the spleen) in the upper chamber of the transwell for 48 h. For polarization analysis, 1 × 10^5^ macrophages (CD11b^+^, isolated from mouse liver) were seeded in DMEM high glucose medium (Solarbio Life Sciences) with 10% FBS. The indicated concentrations of IL‐10 and Ccl2 (Novoprotein, Shanghai, China) were used to stimulate the cells for 6 h.

### ELISA Analysis and Serum Alanine Aminotransferase (ALT) Detection

Hepatic Ccl2, IL‐6, and TNF‐α levels were quantified using a mouse Quantikine ELISA kit (RD Systems, Minneapolis, USA) according to the manufacturer's instructions. The optical density (OD) of the samples was measured with a Varioskan Flash (Thermo Fisher Scientific, Shanghai, China) microplate reader. For ALT detection, peripheral blood samples were analyzed using the UniCel DxC 800 Synchron Clinical System (Beckman Coulter GmbH, Brea, USA).

### Western Blotting

Liver samples were collected and sonicated in RIPA buffer (Solarbio Life Sciences) supplemented with a protease inhibitor cocktail (Bimake, Shanghai, China). The total protein concentration was determined using a BCA Protein Assay Kit (Solarbio Life Sciences). Twenty micrograms of protein were separated on an SDS‐polyacrylamide gel and transferred to PVDF membranes (LABLEAD, Beijing, China), followed by blocking with skimmed milk (OXOID, Hants, UK). Membranes were probed with primary antibodies and incubated with HRP‐conjugated secondary antibodies. Chemiluminescence detection systems (GE Healthcare Life Sciences, Pittsburgh, USA) were used to visualize the protein bands. The antibodies used were as follows: Cyclin D1 (Cell Signaling Technology, Cat#55506, 1:1000), Cdk2 (Cell Signaling Technology, Cat#18048, 1:1000), p‐Akt (S473, Solarbio, Cat# K009354P, 1:1000), Akt (Solarbio, Cat# K106557P, 1:1000), p‐Stat3 (S727, Solarbio, K010036P, 1:1000), Stat3 (Solarbio, Cat# K000124M, 1:1000), Phospho‐IRF3 (S396, Huabio, HA722772, 1:1000), IRF3 (Servicebio, GB11368, 1:1000), STING (Servicebio, GB111415, 1:1000), Ccl2 (Servicebio, GB11199, 1:1000), Phospho‐NF‐κB p65 (S468, Proteintech, Cat# 82335, 1:1500), NF‐κB p65 (Proteintech, Cat# 80979, 1:2000), Phospho‐Erk1/2 (Thr202/Tyr204, Proteintech, Cat# 80031, 1:2000), Erk1/2 (Proteintech, Cat# 66192, 1:2000), Tubulin (Cell Signaling Technology, Cat#5666, 1:2000).

### Histology and Immunohistochemistry

For histological analysis, livers were fixed in 4% paraformaldehyde (Macklin Inc., Shanghai, China) and embedded in paraffin. Paraffin‐embedded tissue was sliced and stained with haematoxylin‐eosin. Subsequently, the sections were subjected to a standard process as described previously for immunohistochemistry and immunofluorescence assays.^[^ ^34^ ^]^ Images were acquired using a DM2700 microscope (Leica). The primary antibodies used for immunostaining included Albumin (Huabio, ET1702‐55, 1:1000), Ki‐67 (Servicebio, Cat# GB111141, 1:500), and CD11b (Servicebio, Cat# GB11058, 1:500). A TUNEL Cell Apoptosis Detection Kit (Servicebio, Wuhan, China) was used to detect apoptotic cells.

### Statistical Analysis

Statistical analyses were performed using GraphPad Prism Version 9 (GraphPad). Depending on the normality (Shapiro‐Wilk test) and homogeneity of variance (Brown‐Forsythe test) of the data, various statistical tests were employed, including the unpaired Student's *t*‐test for normally distributed data between two groups, the Mann–Whitney U test for non‐normally distributed data between two groups, one‐way ANOVA with Bonferroni's post hoc test for comparisons among three or more groups with normally distributed data, and two‐way ANOVA with Bonferroni's post hoc test for analyzing the interaction between two independent variables. When the data did not meet the assumptions required for ANOVA, the Kruskal–Wallis test was used as a non‐parametric alternative. In addition, the Spearman's correlation analysis was performed to determine the possible correlation. Wherever possible, the analyses were performed in a blind manner to minimize bias. All statistical tests were conducted in a two‐sided manner with a significance level set at *p* < 0.05. Group data were expressed as mean + SEM. The number of animals used (*n*) in every experiment are provided in figure legends. Detailed statistics are provided in Table S6 (Supporting Information).

## Conflict of Interest

The authors declare no conflict of interest.

## Author Contributions

R.W., Q.L., and Q.Z. contributed equally to this work. K.W. and P.Y. conceived and designed the study; R.W., Q.L., Q.Z., S.Z., Y.L., B.L., Q.F., X.B., Y.M., X.M., and B.C. performed the experiments; N.W. and P.Y. analyzed the sequencing data; Y.Z. and K.W. wrote the manuscript.

## Supporting information



Supporting Information

Supplemental Table 1

Supplemental Table 2

Supplemental Table 3

Supplemental Table 4

Supplemental Table 5

Supplemental Table 6

## Data Availability

The data that support the findings of this study are openly available in [NGDC GSA database] at [https://www.bigd.big.ac.cn/gsa], reference number [CRA003280 and CRA008215].
