# Supplementary material for: Ccl2‐Induced Regulatory T Cells Balance Inflammation Through Macrophage Polarization During Liver Reconstitution
Source: Adv Sci (Weinh). 2024 Oct 1;11(45):2403849. doi: 10.1002/advs.202403849 (PMC11615773; doi:10.1002/advs.202403849)
Supplement: Supplementary file 1 — Supporting Information [file ADVS-11-2403849-s003.docx]

**Ccl2-induced Regulatory T Cells Balance Inflammation through Macrophage Polarization during Liver Reconstitution**

**Supplementary Figures**

Supplementary Figure S1. Liver injury leads to an abnormal immune status.

Supplementary Figure S2. ILC1s is involving in liver regeneration.

Supplementary Figure S3. RNA-seq analysis of ILC1s in regenerative liver 6 hour after PHx.

Supplementary Figure S4. Ccr2/Ccl2 axis recruits Tregs homing to the injured liver.

Supplementary Figure S5. Deficiency of Tregs shows an enhanced regenerative proliferation after PHx.

Supplementary Figure S6. Identifying T cell subtypes by scRNA-seq data.

Supplementary Figure S7. Tregs or IL-10 treatment has no direct effect on hepatocyte proliferation.

Supplementary Figure S8. Identifying macrophage change during liver injury after scRNA-seq data.

Supplementary Figure S9. Treg-derived IL-10 regulate macrophage polarization.

**Supplementary Figure S1**

**
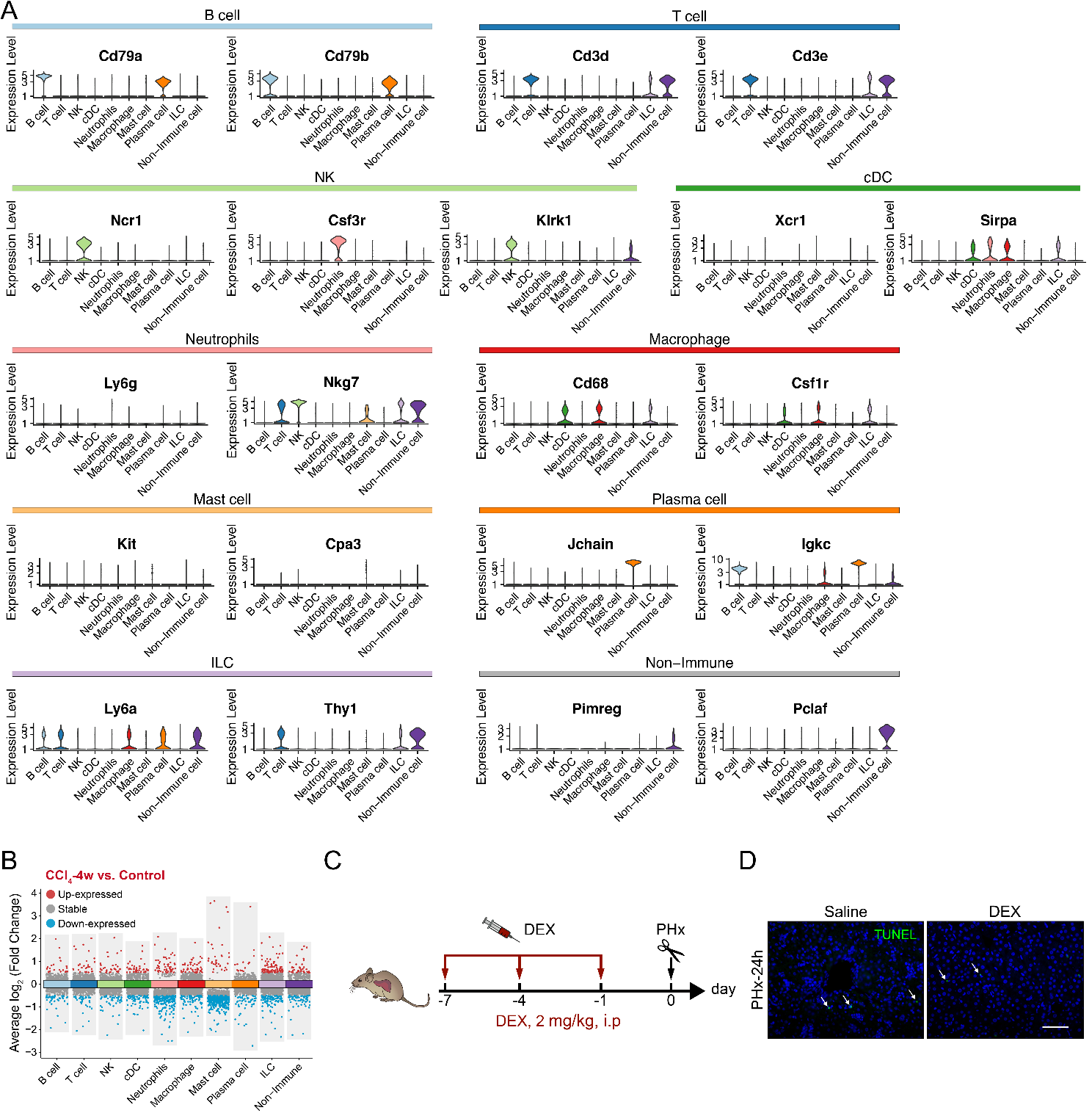
Supplementary Figure S1. Liver injury leads to an abnormal immune status.** (A) The expression of marker genes in different cell subsets by scRNA-seq (*n* = 3). (B) The expression of differentially expressed genes (DEGs) among ten major cell type (4 weeks vs. control, *n* = 3). (C) Flow chart of DEX administration. (D) Representative images of TUNEL staining 24 h after PHx (*n* = 5, scale bar, 100 μm). Data represent three independent experiments.

**Supplementary Figure S2**

**
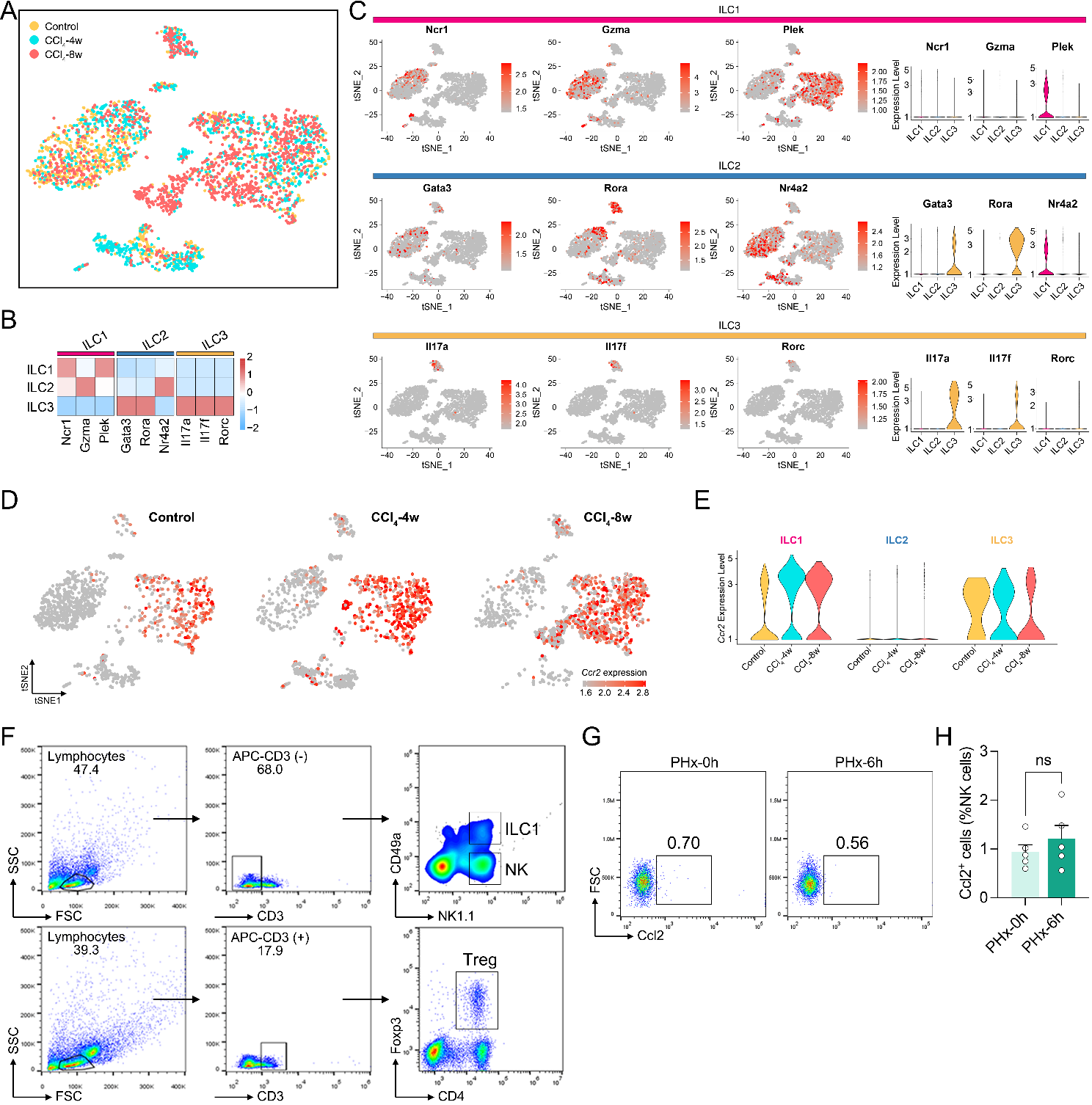
Supplementary Figure S2.** **ILC1s is involving in liver regeneration.** (A) *t*SNE clustering of ILC showing different phase after CCl_4_ treatment by scRNA-seq (*n* = 3). (B and C) The expression of marker genes in ILC subsets by scRNA-seq (*n* = 3). (D and E) The specificity (D) and intensity (E) of *Ccr2* expression in ILC subtypes (*n* = 3). (F) Gating strategy defining the ILC1, NK, and Treg populations. (G and H) Representative flow cytometry plots (G) and percentage (H) of the Ccl2^+^ cells in NK (gated from NK1.1^+^ CD49^-^ cells) (*n* = 5). Data represent three independent experiments. Data are shown as the mean + SEM along with individual data points and were compared using unpaired Student's *t*-test (H). ns indicates *p* > 0.05.

**Supplementary Figure S3**

**
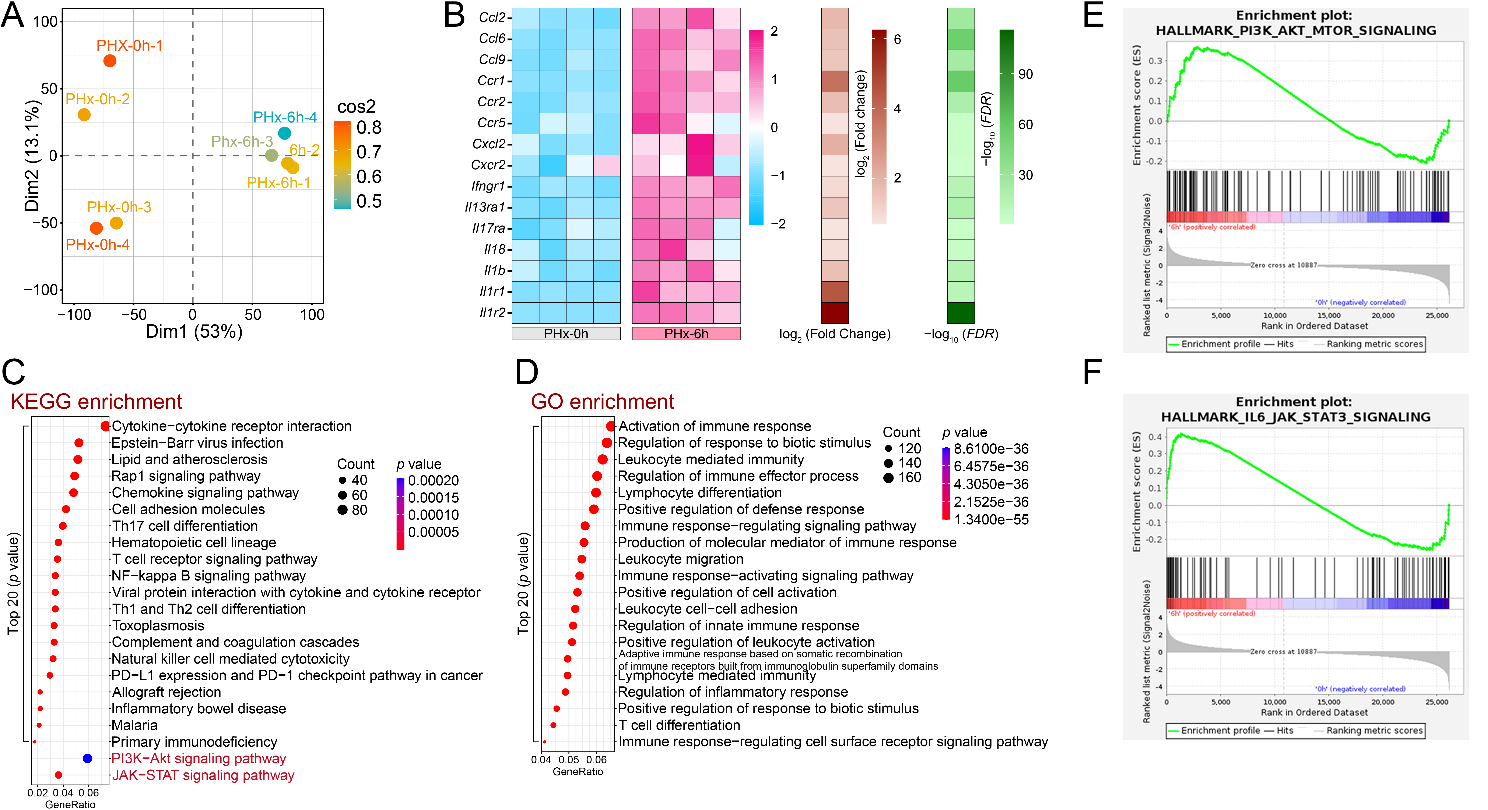
Supplementary Figure S3. RNA-seq analysis of ILC1s in regenerative liver 6 h after PHx.** (A) Principal component analysis (PCA) of transcriptome in ILC1s 6 h after PHx by bulk RNA-seq (*n* = 4). (B) Heatmap of partial DEGs in ILC1s (*n* = 4). (C and D) KEGG (C) and GO (D) analysis of the differentially expressed genes (*n* = 4). (E and F) Gene set enrichment analysis showing that the differentially expressed genes of PI3K/AKT (E) and IL-6/STAT3 (F) signaling (*n* = 4).

**Supplementary Figure S4**

**
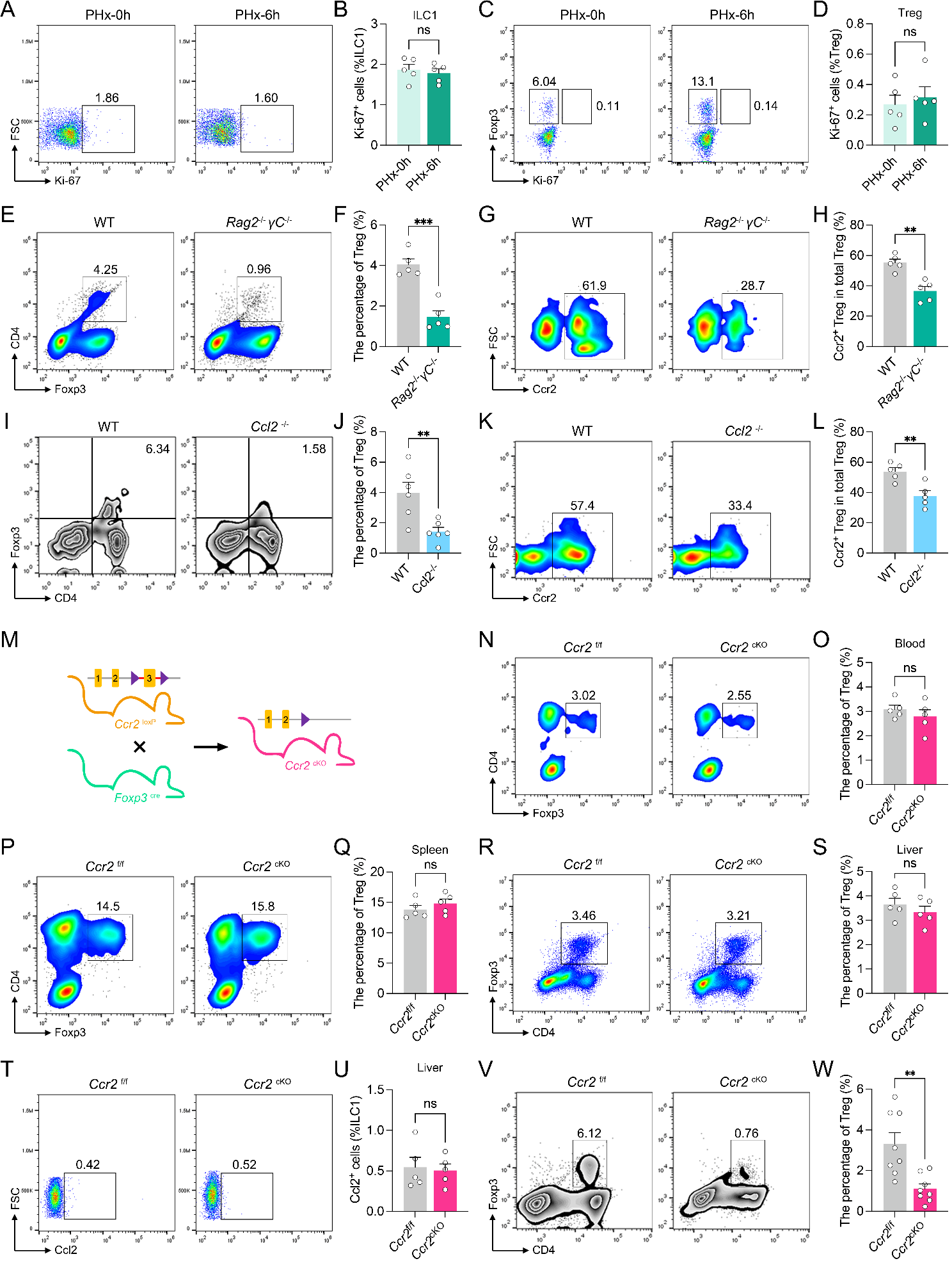
Supplementary Figure S4.** **Ccr2/Ccl2 axis recruits Tregs homing to the injured liver.** (A and B) Representative flow cytometry plots (A) and the statistical quantification (B) of hepatic Ki-67^+^ ILC1s (gated from NK1.1^+^ CD49^+^ cells) before and after PHx (*n* = 5). (C and D) Representative flow cytometry plots (C) and the statistical quantification (D) of hepatic Ki-67^+^ Tregs (gated from CD4^+^ T cell) before and after PHx (*n* = 5). (E-H) Representative flow cytometry plots (E and G) and the statistical quantification (F and H) of hepatic total and Ccr2^+^ Tregs 6 h after PHx in *Rag2*^-/-^*γC*^-/-^ mice (*n* = 5). (I-L) Representative flow cytometry plots (I and K) and the statistical quantification (J and L) of hepatic total and Ccr2^+^ Tregs 6 h after PHx in *Ccl2*^-/-^ mice (*n* = 5, 6). (M) Schematic of generation of *Ccr2*^cKO^ mice. (N-S) Representative flow cytometry plots and the statistical quantification of Tregs in blood (N and O), spleen (P and Q) and liver (R and S) of normal *Ccr2*^cKO^ mice (*n* = 5). (T and U) Representative flow cytometry plots (T) and the statistical quantification (U) of Ccl2^+^ ILC1s in liver of normal *Ccr2*^cKO^ mice (*n* = 5). (V and W) Representative flow cytometry plots (V) and the statistical quantification (W) of hepatic Tregs 6 h after PHx (*n* = 8). Data represent three independent experiments. All data are shown as the mean + SEM along with individual data points and were compared using unpaired Student's *t*-test. ^**^*p* < 0.01, ^***^*p* < 0.001, ns indicates *p* > 0.05.

**Supplementary Figure S5**

**
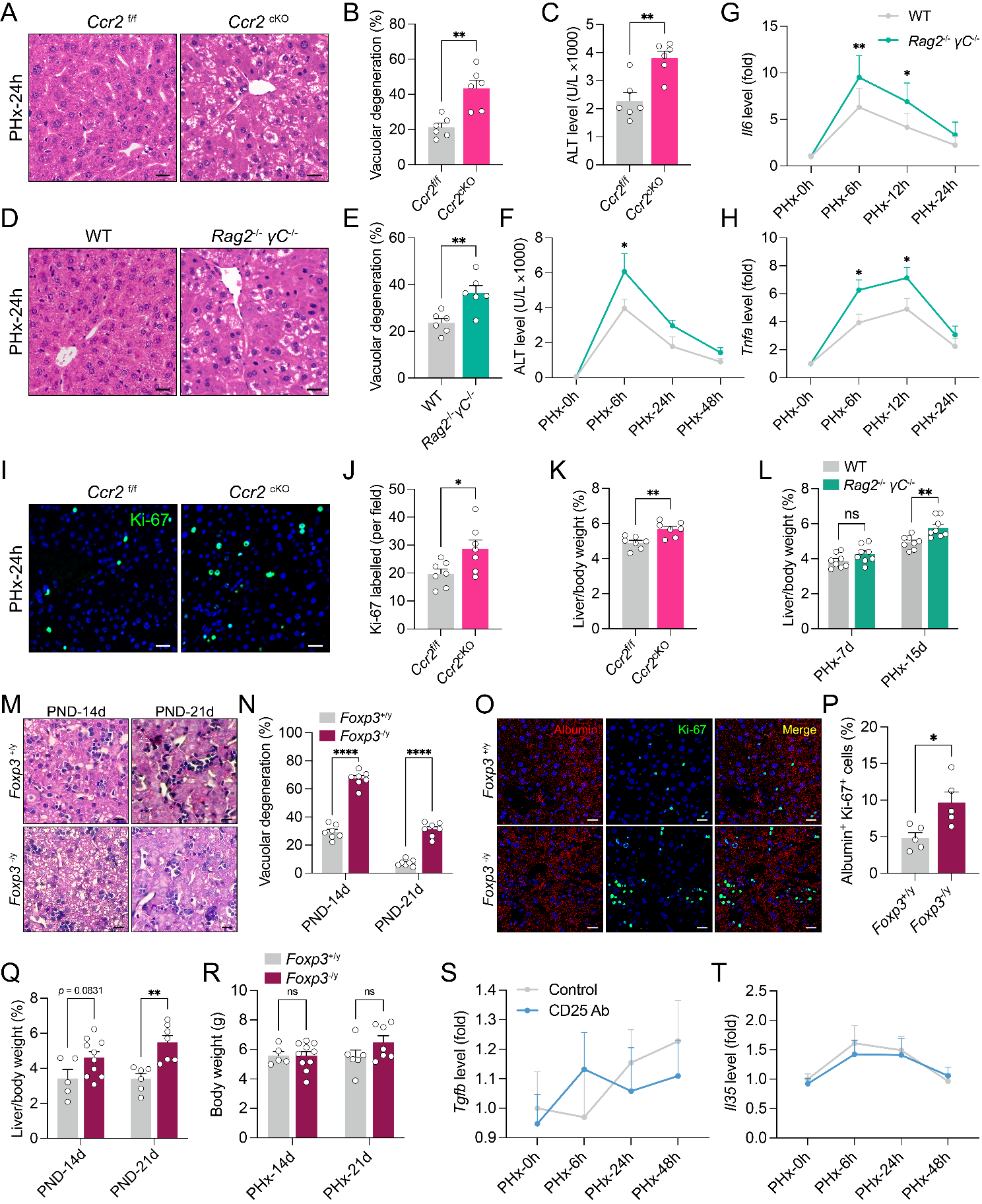
Supplementary Figure S5.** ***Ccr2*^cKO^ mice show an enhanced regenerative proliferation after PHx.** (A and B) Representative images of HE staining (A) and quantification (B) of vacuolar degeneration 24 h after PHx in *Ccr2*^cKO^ mice (*n* = 6, scale bar, 20 μm). (C) Serum levels of ALT after PHx in *Ccr2*^cKO^ mice (*n* = 6). (D and E) Representative images of HE staining (D) and quantification (E) of vacuolar degeneration 24 h after PHx in *Rag2*^-/-^*γC*^-/-^ mice (*n* = 6, scale bar, 20 μm). (F) Serum levels of ALT after PHx in *Rag2*^-/-^*γC*^-/-^mice (*n* = 5). (G and H) The relative mRNA expression of *Il6* (G) and *Tnfa* (H) after PHx in *Rag2*^-/-^*γC*^-/-^ mice (*n* = 5). (I and J) Representative fluorescence images (I) and quantification (J) of Ki-67-positive cells 24 h after PHx in *Ccr2*^cKO^ mice (*n* = 7, scale bar, 20 μm). (K and L) The ratio of liver/body weight in *Ccr2*^cKO^ (K) and *Rag2*^-/-^*γC*^-/-^ (L) mice (*n* = 7, 8). (M and N) The liver tissue from control (*Foxp3*^+/y^) and scurfy (*Foxp3*^-/y^) mice was collected at postnatal days 14 and 21. Representative images of HE staining (M) and quantification (N) of vacuolar degeneration (*n* = 7, scale bar, 20 μm). (O and P) Representative fluorescence images (O) and quantification (P) of Ki-67-positive cells (*n* = 5, scale bar, 20 μm). (Q and R) The ratio of liver/body weight (Q) and the body weight alteration (R) (*n* = 5, 6, 7, 10). (S and T) The relative mRNA expression of *Tgfb* (S) and *Il35* (T) (*n* = 5). Data represent three independent experiments. Data are shown as the mean + SEM along with individual data points and were compared using unpaired Student's *t*-test (B, C, E, J, K and P) or two-way ANOVA followed by Bonferroni's multiple comparisons test (F-H, L, N and Q-T). ^*^*p* < 0.05, ^**^*p* < 0.01, ^****^*p* < 0.0001, ns indicates *p* > 0.05.

**Supplementary Figure S6**

**
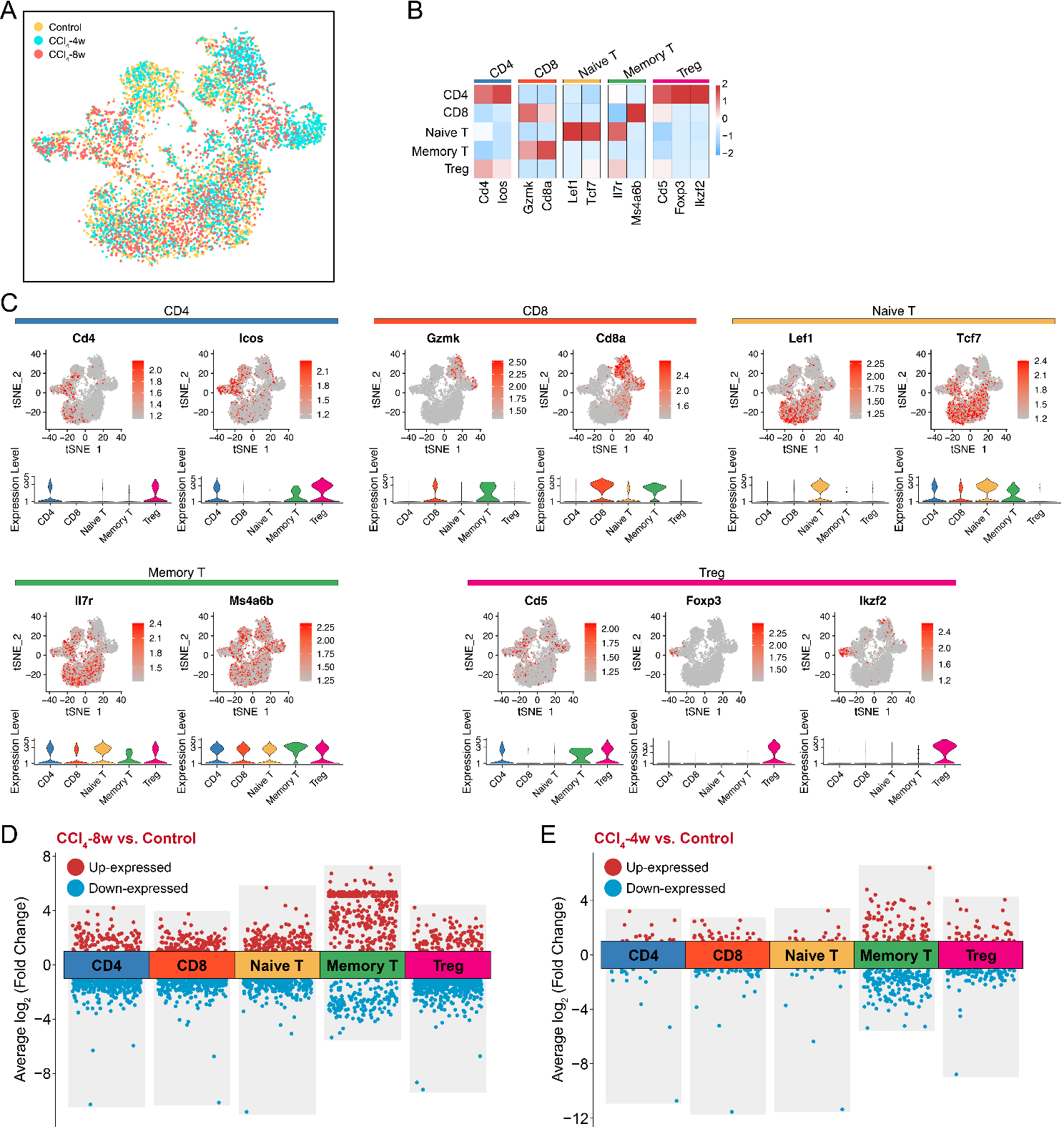
Supplementary Figure S6.** **Identifying T cell subtypes by scRNA-seq data.** (A) *t*SNE clustering showing different phase after CCl_4_ treatment by scRNA-seq (*n* = 3). (B and C) The expression of marker genes in T cell subsets by scRNA-seq (*n* = 3). (D and E) The expression of differentially expressed genes (DEGs) among T cell subsets (*n* = 3).

**Supplementary Figure S7**

**
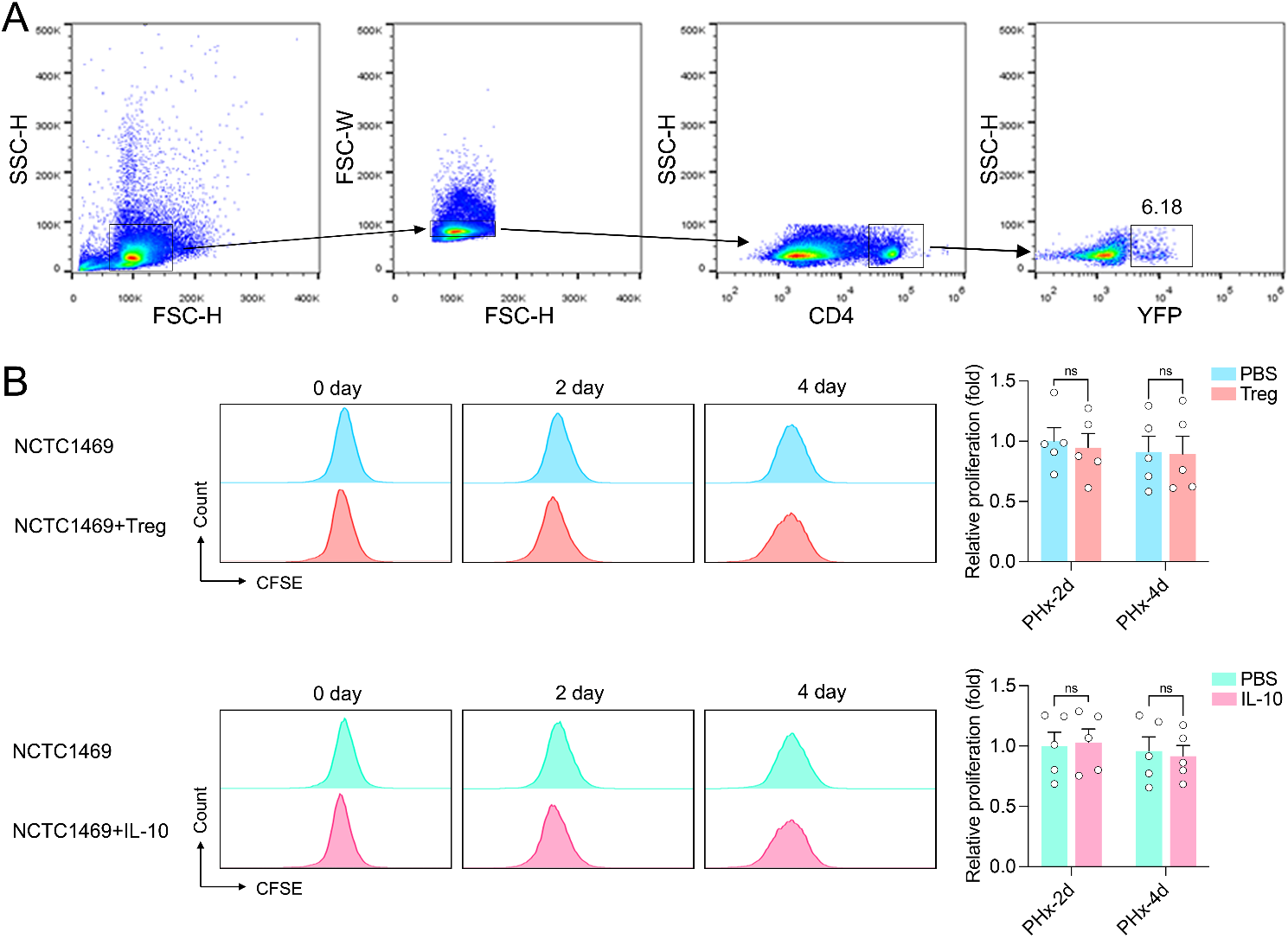
Supplementary Figure S7.** **Tregs or IL-10 treatment has no direct effect on hepatocyte proliferation.** (A) Gating strategy defining the Treg population in *Foxp3*^YFP-Cre^ mice. (B) NCTC1469 cells were labelled with CFSE and treated with PBS/IL-10 for 2 days or 4 days. Representative histogram plots and quantification of the proliferation ratio of NCTC1469 cells (*n* = 5). Data represent three independent experiments. All data are shown as the mean + SEM along with individual data points and were compared using two-way ANOVA. ns indicates *p* > 0.05.

**Supplementary Figure S8**

**
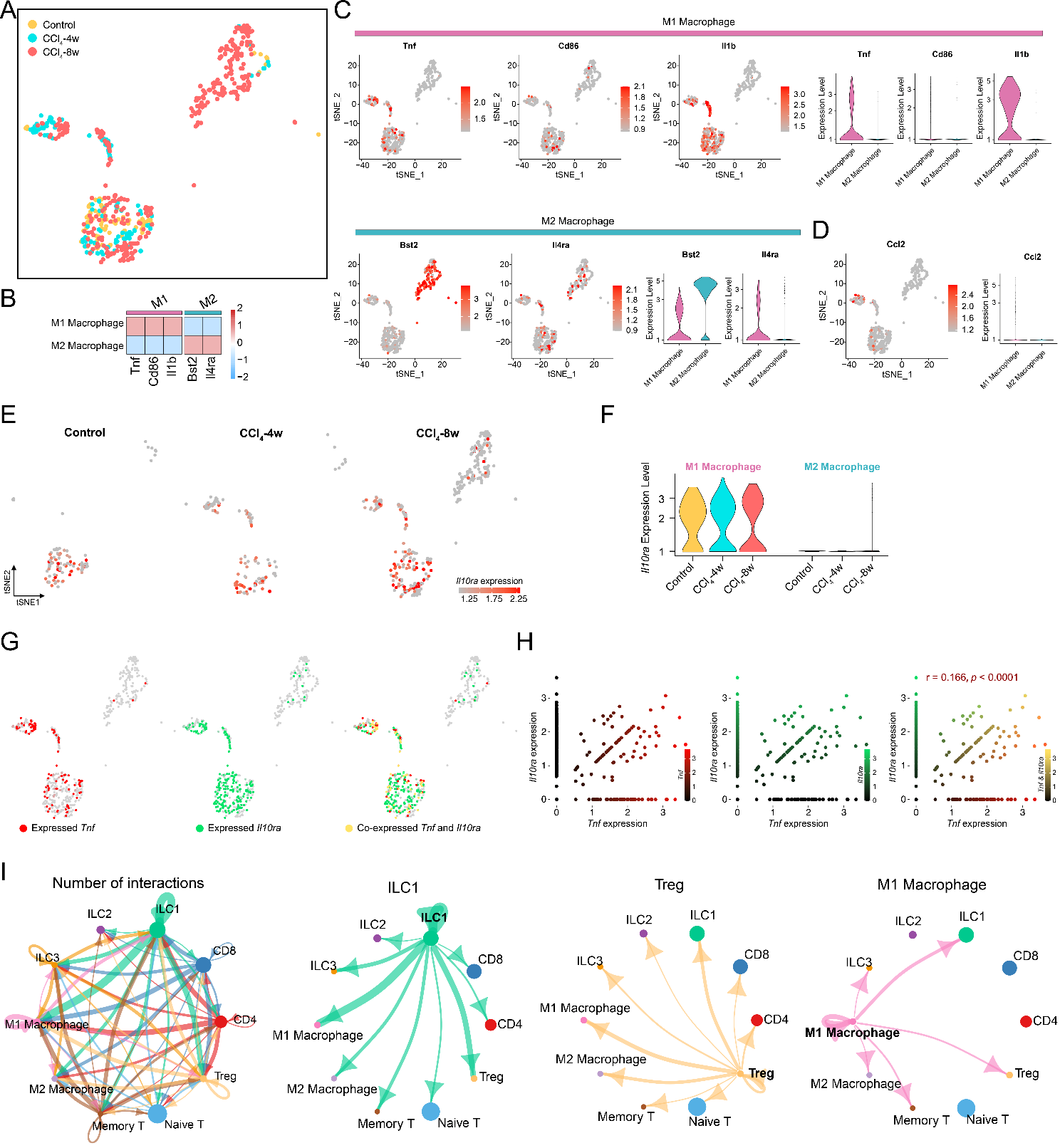
Supplementary Figure S8.** **Identifying macrophage change during liver injury after scRNA-seq data.** (A) *t*SNE clustering showing different phase after CCl_4_ treatment by scRNA-seq (*n* = 3). (B-C) The expression of marker genes in macrophage subsets by scRNA-seq (*n* = 3). (D) The expression of *Ccl2* in macrophage subsets by scRNA-seq (*n* = 3). (E and F) The specificity (E) and intensity (F) of *Il10ra* expression in macrophage (*n* = 3). (G) The expression of *Tnf* and *Il10ra* in *t*SNE plot. *Tnf*-positive cell colored by red. *Il10ra*-positive cell colored by green. *Tnf* and *Il10ra* coexpression colored by yellow (*n* = 3). (H) Correlation analysis between the *Tnf* and *Il10ra* expression (*n* = 3). (I) CellChat analysis showing the interaction between ILC, Treg, and macrophage (*n* = 3). The Spearman’s correlation analysis was used in panel H.

**Supplementary Figure S9**

**
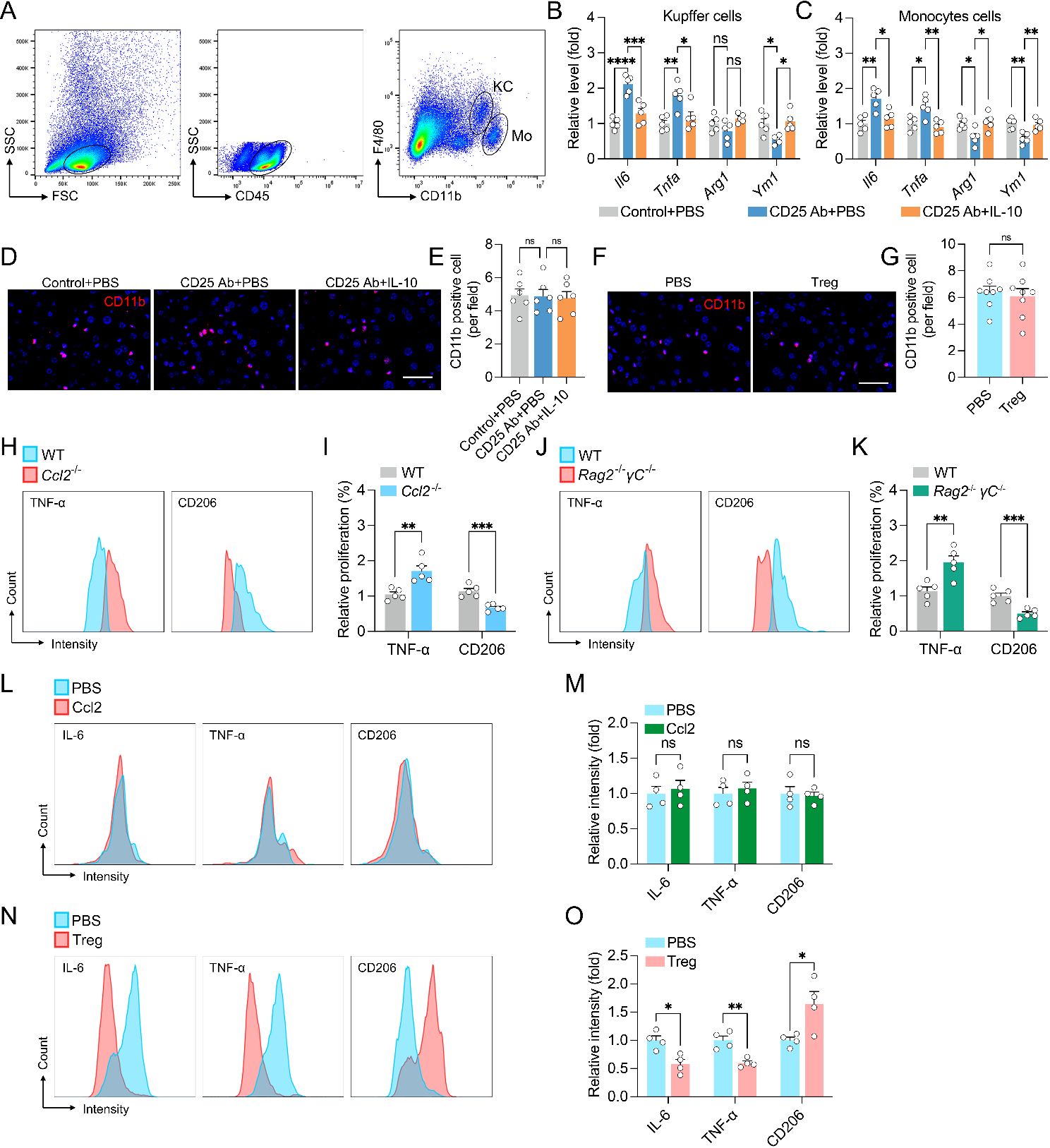
Supplementary Figure S9. Treg-derived IL-10 regulate macrophage polarization.** (A) Gating strategy defining the hepatic total macrophages (CD11b^+^), Kupffer cells (CD11b^+^ F4/80^high^) and monocyte-derived macrophages (CD11b^+^ F4/80^low^). (B and C) Mice received Tregs adoption or anti-CD25 antibody and IL-10 administration as shown in Figure 5A and B. The relative mRNA expression of *Il6*, *Tnfa*, *Arg*, and *Ym1* of in Kupffer cells (B) and monocyte-derived macrophages (C) 24 h after PHx (*n* = 5). (D-G) Representative immunofluorescence images of CD11b staining (D and F) and quantification (E and G) of CD11b-positive cells 6 h after PHx (*n* = 6, 8, scale bar, 50 μm). (H-K) Representative histogram plots (H and J) and quantification (I and K) of the fluorescence intensity of macrophage (CD11b^+^)-gated TNF-α and CD206 24 h after PHx in *Ccl2*^-/-^ and *Rag2*^-/-^*γC*^-/-^ mice (*n* = 5). (L and M) Macrophages were isolated and incubated with 100 ng/ml Ccl2 for 48 h. Representative histogram plots (L) and quantification (M) of the fluorescence intensity of IL-6, TNF-α and CD206 (*n* = 4). (N and O) Tregs and macrophages were isolated and co-cultured in transwell for 48 h. Representative histogram plots (N) and quantification (O) of the fluorescence intensity of IL-6, TNF-α and CD206 (*n* = 4). Data represent three independent experiments. Data are shown as the mean + SEM along with individual data points and were compared using one-way ANOVA followed by Bonferroni's multiple comparisons test (B, C and E) or unpaired Student's *t*-test (G, I, K, M and O). ^*^*p* < 0.05, ^**^*p* < 0.01, ^***^*p* < 0.001, ^****^*p* < 0.0001, ns indicates *p* > 0.05.
